# Determinants of Family Health Performance in Bangladesh: Evidence From MICS‐6

**DOI:** 10.1155/ghe3/3678203

**Published:** 2026-05-30

**Authors:** Robiul Islam Akash, Mohammad Anamul Haque, Nahid Sultana, Mohammad Ohid Ullah

**Affiliations:** ^1^ Department of Statistics, Shahjalal University of Science and Technology, Sylhet, 3114, Bangladesh, sust.edu

## Abstract

**Background:**

Health developments are based on family‐specific health traditions, which play a central role as a foundation of individuals’ growth and development in Southeast Asia. Families can influence disease susceptibility and promote well‐being by encouraging healthy habits, providing support and aiding in disease recovery. Moreover, education, economic conditions, access of hygiene latrines and cooking fuel affect individuals’ health. Few studies have assessed family health as a composite construct in Bangladesh. There is a lack of research on how a family individual’s risk factors contribute to overall family health performance.

**Methods:**

Family‐ and country‐specific data were obtained from Multiple Indicator Cluster Surveys (MICS‐6). MICS data, which were gathered in partnership with UNICEF and the Bangladesh Bureau of Statistics (BBS), are a trustworthy secondary data source. MICS offer statistically reliable and globally comparable statistics. For this study, descriptive statistics tools were used for univariate analysis. Multiple Regression models and Multinomial Logistic Regression models were used for assessing family health performance and identifying factors associated with family health performance.

**Results:**

Data from 24,000 families were used in this study, where 26.33% were from urban areas and 73.67% were from rural areas. The descriptive findings showed that 78.85% of families use traditional cook stoves for cooking their food. Moreover, 25.81% of families have availability for hand washing and 11.61% of children in families face diarrhoea under worst family health category. Furthermore, 59.50% of family head education level is primary or none and 25.79% of families belong to the poorest wealth category. Further, this study developed a family health score to determine which indicators contribute to various family health terms as “Worst family health,” “Average family health,” “Better family health,” and “Excellent family health.” The result of multiple linear regressions and multinomial logistic regression showed that family wealth, types of cook stoves used by families, education level of family head and electricity facilities significantly influence family health performance. This proves that family head education level, family wealth and cook stoves might be influential factors for family health performance.

**Conclusions:**

This study aims to provide insights into family health conditions and identify various significant risk factors. The government must expand educational possibilities, raise public awareness of the value of education and, if at all feasible, mandate that all citizens complete a minimum education. Investments in clean cooking, electricity and education may support to improve family health.

## 1. Introduction

Families have a significant impact on people’s and communities′ well‐being throughout their lives, but efforts to monitor family health trends and investigate their connections to individual and societal outcomes have been hampered by the absence of a validated family health measure [1]. A family’s physical, social, emotional, financial and medical resources, as well as each member’s health, interactions and capacities, all intersect to form family health, which is defined as “a resource at the level of the family unit” [2]. Therefore, the family is the primary unit of social structure in any society, serving as the foundation for individual and community well‐being [3]. In Southeast Asia, where familial bonds are deeply entrenched in social and cultural norms, the household serves as the primary institution for nurturing physical, psychological and social well‐being [4, 5]. Families play a crucial role in low‐resource environments like Bangladesh by mediating access to healthcare, influencing health behaviours and acting as a buffer against socioeconomic and environmental risks [6–8]. The primary objective of this study is to evaluate overall family health performance using the family health score (FHS) and determine the key determinants influencing it.

Existing research highlights the strong link between socioeconomic conditions and fragmented determinants of family health, including poverty [9, 10], parental education [11, 12], sanitation [13–15], household air pollution [16–18] and knowledge on hygiene [19]. For instance, low socioeconomic status (SES) correlates with reduced healthcare access [10], malnutrition [9] and impaired childhood development [7], while parental education improves hygiene practices and caregiving efficacy [9–11], [20]. Environmental factors such as contaminated water and reliance on biomass fuels further compound risks, disproportionately affecting rural households [14, 18]. Yet, these determinants are often studied in isolation, neglecting their synergistic impacts on collective family health. Furthermore, family location and social disparity influence children’s immunization, which is very important for children’s better and healthier lives [20]. Unsafe drinking water is another major cause of death and disease globally. Maintaining cleanliness and hygiene in our houses can have a significant impact on our health conditions. The most common way that diarrhoeal illness spreads is through contaminated water [14]. Moreover, households with improper latrines are associated with morbidity [13]. According to WaterAid Bangladesh Facts and Statistics, in Bangladesh, 70 million (which is almost 41% of the total population) people do not use decent latrine [15]. Additionally, families’ cooking places and air pollution from cooking influence health. The use of clean energy for cooking is more conducive to health than solid energy [16]. Around half of the world’s population and 90% of households in rural areas rely on biomass fuels for energy. Burning biomass in basic or traditional stoves results in incomplete combustion and produces high levels of indoor air pollution. When comparing the group utilizing smoke‐free stoves to those who used traditional stoves, the former group experienced fewer symptoms associated with cooking smoke [18], which indicates that cleaner cooking practices can reduce house air pollution exposure, improve health and save lives [17]. While some studies have explored familial aspects like genetic and skin diseases [21], examining overall family‐specific health performance is a novel undertaking for Bangladesh and other Asian countries.

In this study, an FHS has been developed that takes into account the distribution of health components within households. Regression models are then used to assess the score and see how disparities occur across various family levels. In particular, this study aims to produce an FHS which quantifies overall family health based on key determinants, including SES, education, hygiene and healthcare access. With the evaluation of these factors collectively, the FHS provides a more comprehensive assessment of family health beyond individual health indicators. The study used Multiple Indicator Cluster Surveys (MICS) data, which are widely used to assess the trend in socioeconomic and demographic factors and wealth inequity [22, 23]. The study has identified critical exposures influencing family health, facilitating evidence‐based interventions to improve health outcomes across diverse socioeconomic groups. The findings hold implications for global health strategies aiming to achieve equitable outcomes in resource‐limited settings. By bridging the gap between individual and household health research, this study aims to enhance healthcare accessibility and effectiveness, regardless of economic status or external health crises.

## 2. Methodology

### 2.1. Data

This study used MICS6 that provides data relevant to families for different countries. The “household,” “women,” and “children” information files were selected from among the several data files included in the MICS6 data file. This survey offers statistically reliable and globally comparable statistics that are crucial for creating programs and policies that are supported by facts, as well as for tracking the fulfilment of international commitments and national goals.

### 2.2. FHS

To identify specific family health categories, first, an FHS was developed. The score was generated by assigning points to survey variables based on their evaluation, which allows us to identify each family’s health quality. Factors for the FHS were selected based on the social determinants of health framework, specifically targeting three functional domains:

• Environmental hygiene: water source, toilet type and cooking location
• Behavioural practices: handwashing and soap usage
• Maternal/child health outcomes: morbidity history and antenatal care‐seeking

Each variable was chosen based on its validated impact on health outcomes in developing contexts, as supported by guidelines [21, 24, 25]. The relative risk intensity of various parameters was reflected using a hierarchical weighting technique. For instance, infrastructure‐level sanitation serves as a main barrier against community‐level disease transmission, while behavioural qualities serve as a secondary one, sanitation facilities were given greater ordinal weights (1–3) than binary hygiene behaviours (0–1). Water filters are an important element for better health, that is why we give one point to those families that have a water filter in the house. The place of cooking also influences family members’ health. Families that prepare meals outside of their homes were given one point because cooking indoors has a negative impact on health; other households receive zero points.

Drinking water is very important for humans and all animals [24]. Families that obtain drinking water from a better source or who consume UNICEF‐recommended improved drinking water receive one point; otherwise, they receive zero. As toilet type has a significant effect on health [25], we award three points to households that use high‐quality toilet facilities, two points to households that use ordinary toilet facilities and one point to households that use unsanitary toilet facilities. Hand washing is another important factor for healthy life [21]. We awarded one point to homes with access to water for hand washing and zero to those without. In a similar vein, households that wash their hands with soap received one point, while those that did not received zero.

A family member’s illness is described by variables such as youngsters who have diarrhoea, coughed in the last 2 weeks, have chest or nasal issues, have acute respiratory syndrome or have had a fever in the last 2 weeks. Families without a history of certain ailments received one point, those with illness issues received zero points and everyone else received one point. Every family encounters challenges when a member becomes pregnant. We award three points to families that take their pregnant women to a government medical hospital, two points to those who take them to a private hospital and one point to those who don’t take them to any hospital because better families typically take their pregnant women to medical centres for lower complexity and risk. After giving a weighted point, the sum of this point was calculated and the sum was divided by total maximum point, which was denoted by FHS [21]. Eventually, a dependent variable was formed, termed as family kinds, using this FHS. Here, a family can get a maximum of 19 points and a minimum of zero points. The FHS was calculated using the following formula ignoring the null value for all variables:

(1) Si=∑x=1kwxi∑x=1kmaxwx∗100,

where

 
*S*_*i*_ = FHS of *i* th family,
 
*W*_*x* *i*_ = points for every variable in a family,
 max(*W*_*x*_) = maximum points for every variable in a family.

A standardized score between 0 and 100 is thus generated, with higher numbers denoting better family health. The process adheres to accepted composite‐index normalization and building techniques. The Family Health Scale [1, 26] and methodological guidelines on composite indices [27] are cited for precedent and validation of family‐level measurements.

We performed internal consistency testing to make sure the FHS was resilient, and the results showed a Cronbach’s alpha of [0.72], demonstrating dependable scale integration. Additionally, the indicator weights were varied by ±10% for sensitivity analysis; the high correlation (> 0.90) between the original and adjusted scores demonstrated that the index is stable and not unduly affected by slight weight changes.

### 2.3. Multiple Linear Regression Model

A multiple linear regression model was used to determine which factors have a linear impact on the continuous dependent variable FHS. Prior to analysis, the assumptions of normality, linearity and homoscedasticity were verified via residual plots. Multicollinearity was assessed using variance inflation factors (VIF), with all values falling below the threshold of 5.

### 2.4. Multinomial Logistic Regression Model

To apply the multinomial logistic regression model, the FHS was categorized into four categories (Table 1) using percentile value of FHS. In the multinomial logistic regression model, our reference category is “Worst family health.” The categorization is shown in Table 1.

**TABLE 1:** Classification of the continuous dependent variable FHS.

Percentile range	Family category
Health score ≤ 5 percentile	Worst family health (0)
5 percentile < health score ≤ 50 percentile	Avg. family health (1)
50 percentile < health score < 95 percentile	Better family health (2)
Health score ≥ 95 percentile	Excellent family health (3)

We ensured the absence of multicollinearity among predictors and verified that the data contained no significant outliers that could bias the log‐odds. Finally, we encountered missing values in the secondary data, which we addressed by replacing them with the median value for categorical categories. There is a possibility to use multiple imputation in this scenario; however, this is suitable for continuous outcome variable.

## 3. Results

### 3.1. Descriptive Analysis

**FIGURE 1:**
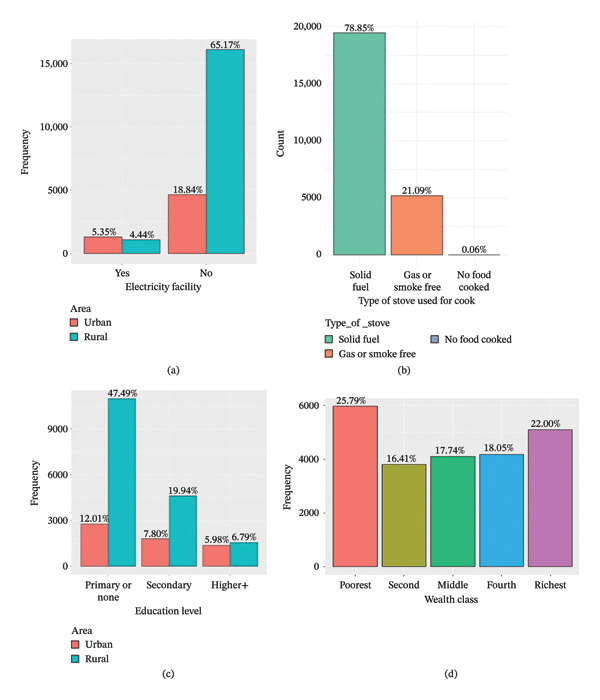
(a) Electricity facilities in house. (b) Cook stoves type used in house. (c) Family head education level. (d) Family wealth index percentage.

Figure 1 displays an exploratory analysis utilizing a number of variables, including the type of cook stoves used in the home, the family wealth index percentage, the family head’s educational attainment and the availability of electricity in the home.

From Figure 1(a), it was observed that the proportion of the families enjoying access to electricity facilities stands at a mere 9.79%, while 90.21% of the families are reportedly deprived of such amenities. The bar chart (Figure 1(b)) provides insights into the types of stoves used for cooking food in families. Solid fuel stoves are known to have bad effects on health compared to other stoves. The data show that a significant proportion of the families, around 78.85%, continue to use traditional solid fuel stoves. According to Figure 1(c), the majority of families’ heads either have not completed primary education or have no formal education at all, which accounts for almost 59.49%. While the majority of families (47.49%) are from rural areas, only 12.01% of the 59.49% of family heads are from urban areas. From Figure 1(d), it was observed that the vast majority of people and families, 25.79% of all families, fall into the lowest wealth index category.

**FIGURE 2:**
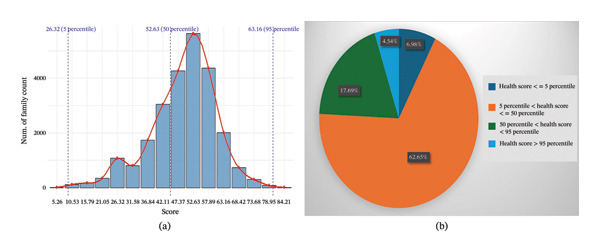
(a) Family health score frequency distribution. (b) Percentage of family health category.

The distribution of FHS is shown in Figure 2(a). It is clear that FHS follows normal distribution. The majority of families are clustered around the median FHS of 52.63, which suggests that most have a medium level of family health.

From Figure 2(b), it can be observed that the FHS of 62.65% falls into the average family health category (5 percentile < Health score ≤ 50 percentile), which is rather typical in Bangladesh.

**FIGURE 3:**
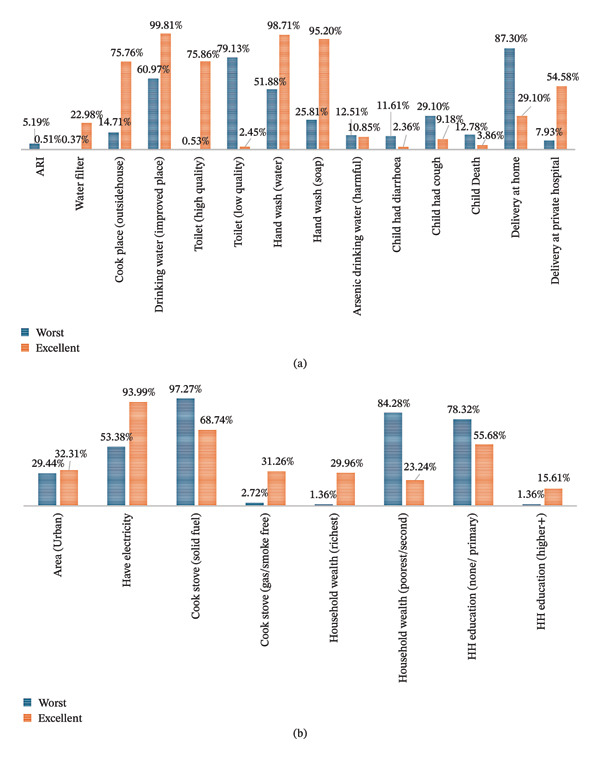
(a) Comparison of dependent variables in “worst” and “excellent” family health category. (b) Comparison of independent variables in “worst” and “excellent” family health category.

Figures 3(a) and 3(b) describe the characteristics of two types of families: one is the “worst family health” and another with “excellent family health.” In Figure 3(a), we observed that 75.76% of families in the excellent health category cook outside of the house, while only 14.71% of families in the worst health category do so. The graph also highlights the disparity in toilet facilities usage, with 79.13% of the worst health category families using low‐quality toilets, compared to only 0.53% using high‐quality facilities. In terms of diseases such as diarrhoea and cough, the worst health category families are affected to a greater extent, while the excellent health category families have a lower percentage of being affected by these diseases. In Figure 3(b), it can be seen that 29.44% of families in the worst health category live in urban areas, compared to 32.31% in the excellent health category, suggesting that living in urban areas does not necessarily confer additional health benefits. The graph also reveals disparities in access to electricity, with 93.99% of families in the excellent health category having access to electricity and only 53.38% of families in the worst health category. Furthermore, 97.27% of families in the worst health category use solid fuel cook stoves, as opposed to 2.72% using smoke‐free or low‐smoke stoves. In contrast, 68.74% of families in the excellent health category use solid fuel stoves and 31.26% use smoke‐free or low‐smoke stoves. The data also indicate that 84.28% of families in the worst health category belong to the poorest or second wealth quintile and only 23.24% of families in the excellent health category. Additionally, family head education quality is higher in the excellent health category, with 15.61% having higher education, compared to only 1.36% in the worst health category.

### 3.2. Multiple Linear Regression Model

With the FHS as the dependent variable, the multiple linear regression model’s parameter estimations are displayed in Table 2.

**TABLE 2:** Linear relationship between family health scores and several factors.

Variables name	Coefficient (95% CI)
Intercept	45.31^∗∗∗^ (44.38–46.23)
Rural (ref.)	
Urban	−1.84^∗∗∗^ (−2.19 to −1.48)
Household electricity (no) (ref.)	
Household electricity (yes)	1.27^∗∗∗^ (0.75–1.78)
Cook stove (solid fuel) (ref.)	
Cook stove (gas)	1.34^∗∗∗^ (0.64–1.61)
Place of cooking	−4.65 (−10.01––0.70)
HH education (pre‐primary/none) (ref.)	
HH education (secondary)	2.10^∗∗∗^ (1.78–2.41)
HH education (higher+)	2.16^∗∗∗^ (1.70–2.41)
HH wealth (poorest) (ref.)	
HH wealth (second)	2.63^∗∗∗^ (2.12–3.14)
HH wealth (middle)	−2.81^∗∗∗^ (−3.28 to −2.34)
HH wealth (fourth)	6.31^∗∗∗^ (5.79–6.84)
HH wealth (richest)	6.58^∗∗∗^ (5.96–7.21)
Don’t attend ECEP (ref.)	
Attend ECEP	0.75^∗∗^ (0.21–1.30)
Age of children	0.29^∗∗∗^ (0.19–0.40)
Children ever breast fed (no) (ref.)	
Children ever breast fed (yes)	−0.65 (−1.51––0.21)

^∗∗^ *p* value < 0.01.

^∗∗∗^ *p* value < 0.001.

Multiple linear regression model findings show that a family’s place of residence influences their health score. In particular, being in an “urban” area is associated with an average decrease of 1.84 units in the FHS compared to being in a “rural” area, assuming all other variables in the model are held constant (*p* value < 0.01). It was found that having electricity has a beneficial impact on household health scores. FHSs rise on average by 1.27 units when families have electricity compared to those without electricity (*p* value < 0.01). According to the findings, families′ health scores improved when they used gas or smokeless stoves compared to solid fuel stoves. When all other factors are held constant, the average health score of households using gas or smoke‐free stoves rises by 1.34 units (*p* value < 0.001), while the average health score of households not cooking at home falls by 4.65 units (*p* value > 0.05).

The education level of the household (in terms of health literacy) head has a significant influence on the overall health of the family. The FHS increases significantly by 2.10 units (*p* value < 0.001) when the household head’s education level rises from primary or no schooling to finishing secondary school. Higher education levels of the household head result in a 2.16 unit point increase in the FHS (*p* value < 0.001).

Furthermore, household wealth was found to have a significant impact (*p* value < 0.001) on FHSs. It was found that FHSs increase by 2.63 units when family wealth increases from the poorest to the second level. However, when family wealth increases from the poorest to the middle level, the FHS decreases by 2.81 units. On the other hand, when family wealth increases from the poorest to the fourth or richest level, the FHS increases by 6.31 units and 6.58 units, respectively.

This model shows a negative relationship between mother breast feeding practice and FHS, though this relation is not statistically significant. However, a positive association was seen between age of children and health score. When children’s age increases, FHS increases on average 0.29 units (*p* value < 0.001).

### 3.3. Multinomial Logistic Regression

With family kind as the dependent variable, the multinomial logistic regression model’s parameter estimations are displayed in Table 3.

**TABLE 3:** Effect of various factors on family‐specific health performance.

Variables name	Avg. Family health	Better family health	Excellent family health
	OR (95% C.I)	OR (95% C.I)	OR (95% C.I)
(Intercept)	13.97^∗∗∗^ (10–19.51)	1.58^∗^ (1.08–2.3)	1.03 (0.69–1.52)
Rural (ref.)			
Urban	0.47^∗∗∗^ (0.42–0.52)	0.45^∗∗∗^ (0.39–0.51)	0.46^∗∗∗^ (0.40–0.52)
Household electricity (no) (ref.)			
Household electricity (yes)	1.69^∗∗∗^ (1.37–2.09)	1.67^∗∗∗^ (1.31–2.12)	1.6^∗∗∗^ (1.22–2.08)
Cook stove (solid fuel) (ref.)			
Cook stove (gas/smoke‐free)	8.27^∗∗∗^ (4.74–14.41)	7.26^∗∗∗^ (4.14–12.70)	7.97^∗∗∗^ (4.55–13.98)
Place of cooking	0.28 (0.07–1.19)	0.0002 5.6e − 16–8.4e + 07)	0.0001 (5.1e − 20–4.7e + 11)
HH education (primary/none) (ref.)			
HH education (secondary)	8.86^∗∗∗^ (7.23–10.86)	8.92^∗∗∗^ (7.23–11.00)	7.5^∗∗∗^ (6.06–9.28)
HH education (higher+)	35.1^∗∗∗^ (16.71–73.72)	38.23^∗∗∗^ (18.15–80.55)	31.37^∗∗∗^ (14.87–66.19)
HH wealth (poorest) (ref.)			
HH wealth (second)	1.83^∗∗∗^ (1.39–2.43)	2.88^∗∗∗^ (2.13–3.88)	4.06^∗∗∗^ (2.96–5.55)
HH wealth (middle)	0.07^∗∗∗^ (0.06–0.08)	0.14^∗∗∗^ (0.12–0.18)	0.23^∗∗∗^ (0.18–0.29)
HH wealth (fourth)	4.07^∗∗∗^ (2.44–6.78)	15.08^∗∗∗^ (8.97–25.35)	24.94^∗∗∗^ (14.71–42.27)
HH wealth (richest)	4.22^∗∗^ (1.93–9.22)	17.76^∗∗∗^ (8.07–39.08)	23.88^∗∗∗^ (10.78–52.89)
Don’t attend ECEP (ref.)			
Attend ECEP	1.34^∗^ (1.08–1.66)	1.4^∗^ (1.11–1.76)	1.22 (0.96–1.56)
Age of children	1.01 (0.97–1.04)	1.07^∗∗^ (1.03–1.11)	1.05 (1.01–1.06)
Breastfeeding after 6 months (no) (ref.)			
Breastfeeding after 6 months (yes)	0.93 (0.68–1.27)	0.96 (0.68–1.35)	0.85 (0.59–1.21)

Abbreviations: CI—confidence interval; OR—odds ratio.

^∗^ *p* value < 0.05.

^∗∗^ *p* value < 0.01.

^∗∗∗^ *p* value < 0.001.

### 3.4. Average Family Health Performance

Multinomial logistic regression models determine the adjusted odds ratio (OR), which describe the effect of individual independent variable on dependent variable. The multinomial model demonstrates that families living in rural areas are more likely to have average FHSs than those families living in urban areas (OR: 0.47, *p* value < 0.001, C.I: 0.42–0.52). It was observed that families which have electricity in their houses are more likely to have better health scores (avg. family health) than those families which have no electricity facilities in‐house (OR: 1.69, *p* value < 0.001, C.I: 1.37–2.09). It is also noted that families that use a gas stove or smoke‐free stoves for cooking food are more likely to have high average family health than those families that use solid fuel stoves for cooking food (OR: 8.27, *p* value < 0.001, CI: 4.74–14.41).

It was observed that increase in household head education from primary or none to secondary (OR: 8.86, *p* value < 0.001, C.I: 7.23–10.86) and higher (OR: 35.10, *p* value < 0.001, C.I: 16.71–73.72) more likely increases the average health of the family. Families whose wealth is second (OR: 1.83, *p* value < 0.001, C.I: 1.39–2.43), fourth (OR: 4.07, *p* value < 0.001, C.I: 2.44–6.78) or richest (OR: 4.22, *p* value < 0.001, C.I: 1.99–9.22) are more likely to have higher average health than the families whose wealth condition is poorest. However, families whose wealth condition is in the middle category are less likely to belong to the average health situation compared to the poorest health condition (OR: 0.07, *p* value < 0.001, C.I: 0.06–0.08). We also observed that when the age of children in a family increases by 1 year, the odds of “average family health” are 1.01 times higher.

### 3.5. Better Family Health Performance

It was observed that families with electricity facilities are more likely to belong to families that have better family health than those families that have no electricity facilities (OR: 1.67, *p* value < 0.001, C.I: 1.31–2.12). This result also showed that families that use gas stoves or smoke‐free stoves are more likely to increase family health than those families that use solid fuel stoves for cooked food (OR: 7.25, *p* value < 0.001, C.I: 4.14–12.70). It is also noted that, if family head education is increased from primary or none to secondary, the family’s health more likely belongs to better quality (OR: 8.92, *p* value < 0.001, C.I: 7.23–11.00). Similarly, if family head education increased to higher, then the family’s health is more likely to belong to better family health (OR: 38.23, *p* value < 0.001, C.I: 18.15–80.50). In subjects of family wealth, our model revealed that family health belongs to better health more likely for families that have second (OR: 2.88, *p* value < 0.001, C.I: 2.13–3.88), fourth (OR: 15.08, *p* value < 0.001, C.I: 8.97–25.35) or richest (OR: 17.76, *p* value < 0.001, C.I: 8.07–39.08) category wealth than families with poorest category wealth.

### 3.6. Excellent Family Health Performance

In multiple logistic regressions, we observed that families living in urban areas are less likely to have excellent family health than families living in rural areas (OR: 0.46, *p* value < 0.001, C.I: 0.40–0.52). It was also found that families which enjoyed electricity facilities are more likely to have excellent family health than families which have no electricity facilities (OR: 1.60, *p* value < 0.001, C.I: 1.22–2.08). It is also noted that families that use gas stoves or smoke‐free stoves are more likely to have excellent families than families that use solid fuel stoves (OR: 7.97, *p* value < 0.001, C.I: 4.55–13.98). Regarding family head’s education level, we observed that when the family head’s education level increased from primary or none to secondary (OR: 7.5, *p* value < 0.001, C.I: 6.06–9.28) or higher (OR: 31.37, *p* value < 0.001, C.I: 14.87–66.19), families are more likely to have excellent family health. Regarding family wealth index group, it was observed that families with wealth increase from poorest to second are more likely to have excellent family health (OR: 4.06, *p* value < 0.001, C.I: 2.96–5.55). Similar situation was also observed when the family wealth index increases from poorest to fourth (OR: 24.94, *p* value < 0.001, C.I: 14.71–42.27) or richest (OR: 23.88, *p* value < 0.001, C.I: 10.78–52.89), that is, the families are more likely to have excellent family health.

## 4. Discussion

One of the most important factors in fostering general well‐being is each person’s and each family’s health. One of the most important factors in fostering general well‐being is each person’s and each family’s health. Every member of the family depends on the general health of the family. In a developing nation like Bangladesh, the entire family is affected when a family member falls ill. If a family member is sick, for instance, it is said that the family’s health is not very good. By identifying these signs, we were able to create FHS. We employed multinomial logistic regression and multiple linear regression to ascertain the effects of several variables on family health. In our analyses, it was observed that families living in rural areas experienced a positive effect on their health. This implies being in an “Urban” area is associated with an average decrease in the FHS compared to being in a “Rural” area, assuming all other variables in the model are held constant.

Illnesses can arise from various factors, for instance, water, air and environmental pollution. Indoor air pollution by smoke from cook stoves has a significant impact on acute respiratory infections (ARIs) in children and family members. In rural areas, indoor air pollution is lower compared to urban areas, due to natural ventilation in homes [28]. Overall, environmental pollution has a more significant impact on health in urban areas than in rural areas [29]. The presence of electricity in homes has a positive impact on family health. Results from different analyses, including multinomial logistic regression and linear regression, have been consistent. These findings support other studies that suggest electricity access can improve health [30, 31]. Cooking meals is a daily necessity for every family, and there are various methods for doing so, including using solid fuel, gas or electricity. However, burning solid fuels produces a significant amount of smoke and carbon emissions, with 90% of the smoke being carbon monoxide, which poses a serious health risk worldwide. Using stoves that emit low or no smoke can greatly benefit the health of family members [18, 29, 31]. Our analysis also indicates that using gas or smoke‐free cooking stoves has a positive impact on family health.

One important element that might enhance the financial and health circumstances of families is education. Education is essential for enhancing socioeconomic and health conditions in emerging nations like Bangladesh. Additionally, our analysis shows that raising the family head’s educational attainment from primary or no schooling to secondary or higher education has a substantial favourable effect on the family’s health, which is supported by several studies [32, 33]. Education is meaningful in terms of health literacy and/or health seeking behaviour, which is a determinant of health [34]. The wealth of a household or family affects the nutrition of children and family members. Several studies have shown that children from poorer houses tend to be more undernourished than children from wealthier families [35]. Another study has found that poor health conditions are strongly associated with low income or poor wealth [36]. The economic or wealth status of a family also has an impact on recovery from diseases. Our research analysis shows that family wealth conditions affect the health of families. As family wealth increases, family health conditions also improve. Breastfeeding is beneficial for both children and mothers, but sometimes it reveals a negative correlation with children’s health. Those may be due to children being feed other foods for their nutrition after 6 months, which could be contaminated and impact their health [37].

## 5. Conclusions

Health is one of the most important assets for human beings. This study aims to provide insights into family health conditions and the factors that can either improve or worsen them. According to this study, several crucial factors have been identified that significantly impact family health conditions. An FHS was developed based on various health indicators and was used to determine which indicators contribute to “Worst family health,” “Average family health,” “Better family health,” and “Excellent family health” conditions.

Both multiple linear regression and multinomial logistic regression have revealed that access to electricity in families improves FHS. Moreover, using gas or smoke‐free cook stoves is another crucial factor that can help reduce family diseases. In addition, the education level of the family head has a positive influence on reducing health issues, thereby increasing the FHS. Family wealth categories also play a crucial role, a rise of family wealth conditions correlates with improved FHS. Multiple linear regression and multinomial logistic regression models both verified this, which demonstrates the significant influence of these variables in decreasing family diseases and improving FHS. It can be recommended that the primary means of improving FHSs and lowering family illnesses are to increase family members′ knowledge of the importance of using sanitary restrooms, washing their hands often, drinking water from better sources and avoiding drinking water contaminated with arsenic. Enhancing the electrical facilities and smoke‐free cooking facilities like gas or biogas facilities within the home will also contribute to a higher health score. The government must expand educational possibilities, raise public awareness of the value of education and, if at all feasible, mandate that all citizens complete a minimum education. Increasing family wealth presents a considerable challenge; however, it remains an achievable objective. With the steps taken by the government to promote a hardworking and industrious citizenry and create more job opportunities, this problem can be addressed effectively. Investments in clean cooking, electricity and education may support improved family health. Our findings suggest associations that warrant further investigation with longitudinal data.

This study also had some limitations. A secondary MICS6 survey, which was conducted in 2019, was used. We further encountered missing values in this data and this missingness were addressed by replacing them with the median value for categorical variables. There is a possibility to use multiple imputation in this scenario; however, this is suitable for continuous outcome variable. Additionally, due to the use of secondary data, we were unable to incorporate additional health indicators and influencing factors. If primary data are utilized in future research, it would enable closer monitoring of family health performance. Finally, the results need to be interpreted with the fact that although the MICS employs a complex sampling design, the results in this study reflected the study sample rather than the weighted national population.

## Author Contributions

Robiul Islam Akash contributed to data modification, formal data analysis, writing–original draft and writing–review and editing. Mohammad Anamul Haque contributed to conceptualization, data modification, framing the methodology, analysing the data, data validation, writing–original draft, supervision and writing–review and editing. Nahid Sultana contributed to results interpretation and writing–review and editing. Mohammad Ohid Ullah contributed to methodology, data validation, overall supervision and writing–review and editing.

## Funding

The authors received no specific funding for this work.

## Conflicts of Interest

The authors declare no conflicts of interest.

## Data Availability

The data are freely available in MICS database. All MICS files can be found at https://mics.unicef.org/surveys, UNICEF′s online database. The data were not subject to any special access rights for the authors. By directly accessing the data via the above URLs, interested researchers can duplicate the study′s conclusions.
